# The Antioxidant Role of One-Carbon Metabolism on Stroke

**DOI:** 10.3390/antiox9111141

**Published:** 2020-11-17

**Authors:** Kassidy Burgess, Calli Bennett, Hannah Mosnier, Neha Kwatra, Forrest Bethel, Nafisa M. Jadavji

**Affiliations:** 1 College of Veterinary Medicine, Midwestern University, Glendale, AZ 85308, USA; kburgess99@midwestern.edu; 2 Biomedical Sciences Program, Midwestern University, Glendale, AZ 85308, USA; cbennett83@midwestern.edu (C.B.); nkwatra46@midwestern.edu (N.K.); fbethel37@midwestern.edu (F.B.); 3 College of Osteopathic Medicine, Midwestern University, Glendale, AZ 85308, USA; 4 School of Medicine, National University of Ireland Galway, H91 TK33, Ireland; H.Mosnier1@nuigalway.ie; 5 College of Dental Medicine, Midwestern University, Glendale, AZ 85308, USA; 6 Department of Neuroscience, Carleton University, Ottawa, ON K1S 5B6, Canada

**Keywords:** hypoxia, antioxidant, stroke, one-carbon metabolism, methionine, transsulfuration

## Abstract

One-carbon (1C) metabolism is a metabolic network that is centered on folate, a B vitamin; it integrates nutritional signals with biosynthesis, redox homeostasis, and epigenetics. This metabolic pathway also reduces levels of homocysteine, a non-protein amino acid. High levels of homocysteine are linked to increased risk of hypoxic events, such as stroke. Several preclinical studies have suggested that 1C metabolism can impact stroke outcome, but the clinical data are unclear. The objective of this paper was to review preclinical and clinical research to determine whether 1C metabolism has an antioxidant role on stroke. To accomplish the objective, we searched for publications using the following medical subject headings (MeSH) keywords: antioxidants, hypoxia, stroke, homocysteine, one-carbon metabolism, folate, methionine, and dietary supplementation of one-carbon metabolism. Both pre-clinical and clinical studies were retrieved and reviewed. Our review of the literature suggests that deficiencies in 1C play an important role in the onset and outcome of stroke. Dietary supplementation of 1C provides beneficial effects on stroke outcome. For stroke-affected patients or individuals at high risk for stroke, the data suggest that nutritional modifications in addition to other therapies could be incorporated into a treatment plan.

## 1. Introduction

**Figure 1:**
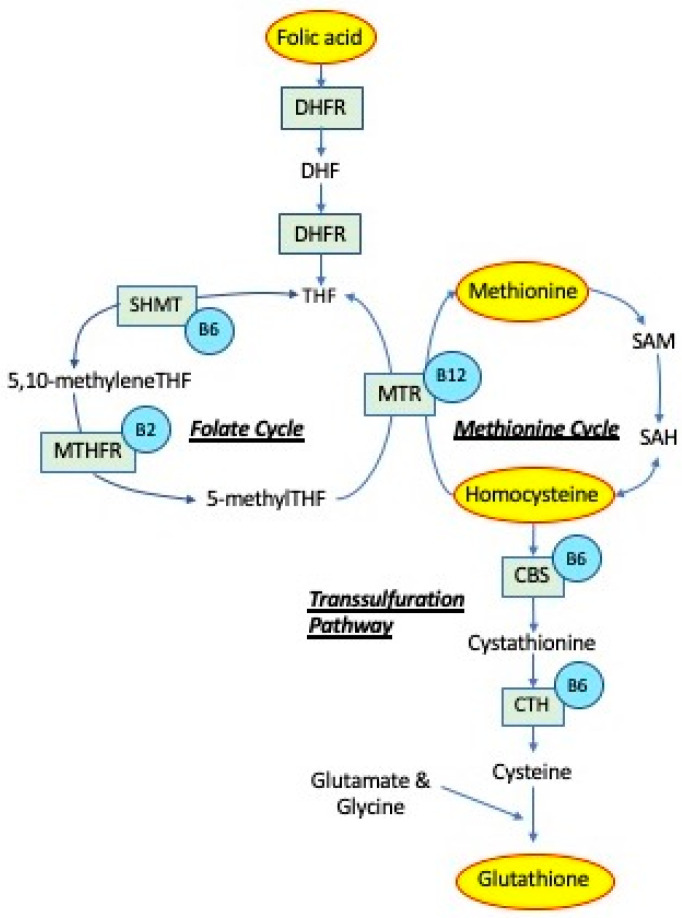
One-carbon metabolism. Folic acid obtained from the diet is reduced to dihydrofolate (DHF) and tetrahydrofolate (THF) by dihydrofolate reductase (DHFR). A methyl group is then transferred to THF by serine hydroxymethyltransferase (SHMT), forming 5,10-methyleneTHF. Vitamin B6 is a cofactor for SHMT. 5,10-methyleneTHF can be reduced by 5,10-methylene tetrahydrofolate reductase (MTHFR) to 5-methylTHF. The methyl group of 5-methyl-THF is transferred to homocysteine by methionine synthase (MTR), generating methionine and regenerating THF. Vitamin B12 is a cofactor for MTR. Methionine is then activated to form S-adenosylmethionine (SAM), which is converted to S-adenosylhomocysteine (SAH) after donating its methyl group. SAH is subsequently hydrolyzed to generate homocysteine. Homocysteine is then used either to regenerate methionine, or homocysteine can be converted to cystathionine by cystathionine beta-synthase (CBS) and further to cysteine by cystathionine gamma-lyase (CTH), using vitamin B6 as cofactor and glutathione, via the transsulfuration pathway.

One-carbon (1C) metabolism is a metabolic network that integrates nutritional signals with biosynthesis, redox homeostasis, and epigenetics, as summarized in Figure 1. It plays an essential role in the regulation of cell proliferation, stress resistance, and embryonic development [1]. The natural form of the B vitamin, folate, is central in 1C, as well as the synthetic form of the vitamin referred to as folic acid. Other vitamins involved in 1C include vitamin B12 and the nutrient choline. In the brain, choline is involved in acetylcholine synthesis and lipid metabolism. Folate and choline metabolism are tightly linked. Choline can act as a 1C donor, especially when there is a deficiency in folate. In the cell, 1C plays an essential role in nucleotide synthesis of purines, removal of uracil from DNA, and methylation, through the metabolism of homocysteine and generation of *S*-adenosylmethionine. Homocysteine can also be metabolized by being pulled into the transsulfuration pathway to generate glutathione.

Several clinical studies have shown that elevations in plasma homocysteine are associated with an increased risk of atherosclerosis and hypoxic events, such as stroke [2,3,4]. The hypothesis that homocysteine may lead to hypoxia was first recognized in 1969 in homocystinuric children with vascular disease [5]. Since this discovery, several studies have demonstrated that homocysteine levels are significantly higher in individuals with multiple stenotic sites within their vasculature [6,7]. Additionally, a prospective study of 5661 men in the UK found that total homocysteine (tHcy) was predictive of increased stroke risk [8]. Many other investigations have observed similar results and found moderate levels of tHcy (9–15 μmol/L) to be associated with ischemic stroke risk independent of sex, ethnicity, and various risk factors associated with stroke [3,9,10]. A 25% decrease in the usual tHcy level has been associated with a 19% lower stroke risk [11]. In mice that were deficient in low-density lipoprotein, increased homocysteine stimulated the dedifferentiation of vascular smooth muscle cells, causing the state of atherosclerosis [12].

Endothelial injury and dysfunction are the leading mechanisms contributing to the development of hypoxia observed in hyperhomocysteinemia. Homocysteine-induced depletion of bioactive nitric oxide (NO), the endothelium-derived factor responsible for vasodilation, has been suggested for the endothelial dysfunction leading to hypoxia [13]. As shown in microvasculature from murine hearts, homocysteine inhibits dimethylarginine dimethylaminohydrolase activity, resulting in the accumulation of the e-NOS (nitric oxide synthase) inhibitor preventing the production of NO [14,15]. Studies using bovine aortic endothelial cells have shown that homocysteine impairs the transportation of L-arginine, a precursor of NO, into endothelial cells, leading to the enzymatic uncoupling of endothelial nitric oxide synthase (e-NOS) [16,17]. The uncoupling of e-NOS causes it to produce superoxide, which can then react with any remaining NO, producing peroxynitrite. A model using murine aortas showed peroxynitrite depletes the cofactor of the e-NOS domain, diverting it from a NO-producing enzyme to a producer of reactive oxygen species (ROS), which could, in turn, trigger a vicious cycle of further e-NOS uncoupling [18,19,20]. A study performed on the cerebral cortex of rats showed additional superoxide can be generated from copper-catalyzed oxidation of homocysteine’s thiol group, inhibiting NO-related cerebrovascular responses [21].

The autoxidation of homocysteine can also produce ROS, and this can induce endothelial damage via oxidative stress, which is considered to be the first step of atherogenesis [22,23]. In addition to increased production of ROS, homocysteine has been shown in bovine aortic endothelial cells (BAECs) in vitro to reduce the intracellular antioxidant enzyme, glutathione peroxidase, thereby potentiating endothelial damage and the inactivation of NO by ROS [24]. As shown in cultured human and bovine endothelial cells, the ROS can directly damage the vessel endothelium, activating the coagulation cascade in an already prothrombotic environment due to enhanced coagulation and impaired fibrinolysis induced by hyperhomocysteinemia [25,26,27]. Furthermore, ROS have been reported to initiate lipid peroxidation of low-density lipoproteins, inducing platelet activation in vitro and exerting an atherogenic effect [28,29]. In addition, excess homocysteine leads to the generation of homocysteine-thiolactone, which can impair the structure and function of proteins, potentially triggering ER stress-related endothelial detachment and apoptosis [30,31,32]. It is worthwhile to note that the concentrations of homocysteine used in some model system studies are far higher than those reported in human plasma (5 to 15 μM) [33].

The remethylation of homocysteine leads to the generation of methionine and then *S*-adenosylmethionine, a global methyl donor. Elevated levels of homocysteine have been shown to target DNA methylation patterns, altering gene expression and creating endothelial cells that are more vulnerable to damage and have an altered response to hypoxia [4,34,35]. In addition, these alterations in gene expression are believed to contribute to atherogenesis [36]. Homocysteine also inhibited DNA synthesis in human vascular endothelial cells and arrested their growth [37]. In contrast, studies using murine cerebral arterioles found that homocysteine promotes hypertrophy vascular smooth muscle cells, creating a smaller lumen within the vessels [38,39]. In summary, deficiencies in 1C metabolism impact vasculature in the body.

The objective of this paper is to review preclinical and clinical research to determine whether one-carbon metabolism has an antioxidant impact on stroke. We searched for publications using the following medical subject headings (MeSH) keywords; antioxidants, hypoxia, stroke, homocysteine, one-carbon metabolism, folate, methionine, and dietary supplementation of one-carbon metabolism. We have reviewed four components of 1C, and these areas include folates/folic acid, dietary supplementation of 1C, methionine, and transsulfuration. Each section includes a review of clinical and preclinical studies. The main findings from all preclinical studies reviewed are summarized in Table 1.

## 2. Folates

A diet deficient in folate can directly cause various biological complications related to the deficiency itself, but can additionally cause indirect damage due to increased homocysteine concentrations [40]. Due to its role in the progression of atherosclerosis, a low folate status can exacerbate the effects of a hypoxic event, such as ischemic stroke. Data collected from ischemic and hemorrhagic stroke patients were used to determine whether stroke risk was affected by folate status. One study reported that increased folate concentrations reduced the risk of hemorrhagic stroke, but the results for ischemic stroke were unclear. The study suggested that, while a low folate status is associated with negative implications related to ischemic stroke, an adequate folate status was not found to be protective against ischemic stroke [41].

After a period of hypoxia, folate concentration can play a role in recovery. A study using rats investigated the mechanisms of folic acid deficiency-induced neuronal injury and found that folate deficiency can promote and aggravate neuronal cell damage after ischemia [42]. The mechanism of this damage was shown to be overactivation of autophagy that was triggered by the deficiency and may also involve oxidative injury [42]. Another study using a gerbil model system reported that a folic acid deficiency can induce early and significant neuronal death through oxidative DNA damage. This demonstrates that folic acid deficiency influences and potentiates the pathological processes that are activated by ischemia [40].

Due to the myriad negative health outcomes that can occur when there is a deficiency in folate, many studies have focused on methods by which to reduce folate deficiencies. It is widely acknowledged that under-provision of folate can be attributed to the poor bioavailability of folate in natural food sources [43]. While naturally occurring folate is labile, its synthetic form, folic acid, is fully oxidized and much more stable. Many factors contribute to the absorption of folate, such as incomplete hydrolysis and destruction of folate in the gastrointestinal tract. However, these factors are mitigated when ingesting the synthetic form because it is more stable and does not require the same cellular machinery to convert it into an active form [44].

While it is understood that folate has a reduced bioavailability (Figure 1), the extent to which it is reduced is controversial [43]. Several studies have demonstrated that folate bioavailability is modulated by factors such as genetic variation and ethnicity/race. For example, the methyl tetrahydrofolate reductase (*MTHFR*) gene, which codes for an essential enzyme in folate metabolism, has a common mutation (*MTHFR* C→T) that has variable occurrence according to location and ethnicity [45]. Even when the *MTHFR* C→T mutation is controlled for, African American women display much lower levels of folate following a diet-controlled study [46]. Due ot these many factors affecting folate bioavailability, folic acid is the preferred compound for supplementation.

## 3. Dietary Supplementation of 1C

1C supplementation, including B vitamins and choline, is an effective method to reduce levels of homocysteine. Since elevated levels of homocysteine lead to increased ROS, downstream oxidative damage has been suggested as a potential mechanism behind homocysteine as a risk factor. Therefore, antioxidant supplementation has been proposed as a method to attenuate stroke risk and improve recovery. In a mouse model study, B vitamin supplementation, along with choline and riboflavin, was demonstrated to increase nuclear factor erythroid 2-related factor 2 (Nrf2) and super oxide dismutase 2 (SOD2), markers of antioxidant activity, after ischemic stroke in a mouse model system [47]. Furthermore, model studies have indicated that supplementation of B vitamins improves functional recovery following an ischemic stroke [47,48]. In addition to functional improvement, folic acid supplementation increased Notch signaling and neurogenesis in the hippocampus [48]. Another model study demonstrated folic acid supplementation leads to increased angiogenesis and neuroprotection after an ischemic event [49]. Additionally, folic acid stabilizes hypoxia inducible factor 1 alpha (HIF-1α), a transcription factor that facilitates cellular adaptation to hypoxia and ischemia [49]. Many model studies have produced promising results, however, benefits become less clear when translated into clinical trials.

Several large-scale clinical trials have evaluated supplementation with folic acid and other B vitamins with supplementation to varying results; we will highlight a few. The Vitamins to Prevent Stroke (VITATOPS) study was a multi-country study investigating potential benefits of folic acid supplementation. A secondary analysis of the study demonstrated that although supplementation reduced levels of homocysteine, it did not have an impact on incidence of cognitive impairment [50]. Additionally, a study conducted in France suggested that B vitamin, omega-3 fatty acid supplementation, or a combination of the two have no significant impact on the prevention of cardiovascular disease for individuals with a history of ischemic heart disease or ischemic stroke [51]. In contrast, the China Stroke Primary Prevention Trial (CSPPT) indicated that folic acid supplementation leads to a significant decrease in incidence of stroke in hypertensive patients when combined with enalapril, an angiotensin-converting enzyme (ACE) inhibitor [52,53]. The CSPPT study indicated that folic acid supplementation is reliable in decreasing the incident of stroke in populations with little to no folic acid fortification programs [54]. However, several meta-data analyses have presented similarly conflicting results [55,56,57].

There could be several possibilities as to why there is a disconnect between the model system and clinical literature. First, it is important to consider where these studies are taking place, and if folic acid fortification is common in those countries or regions. Second, underlying concomitant health factors and drug use of subjects could be significant. A sub-analysis of the CSPPT suggested the effects of folic acid supplementation can be modified based on the patients’ cholesterol levels, with the most significant risk reduction in individuals with high cholesterol [58]. Along a similar vein, statin use has been shown to decrease the effect of folic acid supplementation on stroke risk [54]. A second sub-analysis of the CSPPT data indicated a greatly attenuated impact of folic acid supplementation when patients demonstrated low platelet counts [59]. Additionally, a post hoc analysis of the VITATOPs data indicated that folic acid therapy was modified by anti-platelet therapy [60]. With the conflicting data on the role of 1C supplementation and the myriad factors by which it can be modified, further focused studies are required.

In a clinical setting, folic acid supplementation has been demonstrated to significantly increase the total antioxidant capacity [61]. Additionally, a double-blind placebo-controlled trial on diabetic patients indicated similar effects of folic acid supplementation on total antioxidant capacity [62]. Significantly, higher total antioxidant capacity has been associated with decreased mortality following ischemic stroke [63]. Therefore, it would be logical to assume that with folic acid supplementation leading to increased total antioxidant capacity and increased total antioxidant capacity leading to decreased ischemic stroke mortality, folic acid supplementation would lead to decreased mortality following ischemic stroke.

In model systems studying the impact of reduced levels of dietary folates, it is demonstrated that choline metabolism is also affected [64]. Similar to folates, choline bioavailability is dependent on several factors. It is modulated by the diet and genome via the microbiota of individuals [65]. Additionally, the bioavailability is affected by the structure of the choline source [66]. Further, and also similar to folic acid, several model studies have indicated that choline supplementation following an ischemic event provides neuroprotection. This neuroprotection is especially evident in the hippocampus, including the CA1 pyramidal cells [47,67], and increases cortical sparing following a traumatic brain injury [68]. Components of choline metabolism, including citicoline, have also been examined as potential treatments following an ischemic event. In model studies, citicoline has been demonstrated to restore phospholipid membranes and mitigate oxidative stress following an ischemic event [69]. Additionally, chronic supplementation of citicoline following an ischemic event increased neuroplasticity and functional recovery [70].

## 4. Methionine

Once homocysteine has been remethylated, it will be activated to form methionine (Figure 1) [71]. Methionine is an amino acid that is essential in all animal species for protein [72] and cysteine synthesis [73]. It is involved in 1C through the transmethylation reaction to produce S-adenosylmethionine (SAM) [73]. Several research reports suggest that methionine also plays an important role in the brain during and after hypoxia [74,75,76]. Interestingly, methionine and related derivatives have been shown to protect the brain from the detrimental effects of hypoxia [74,75,76]. The bioavailability of methionine in humans is not well characterized, due to the invasive nature of such studies. There is a study suggesting that the DL-isomer is used at ~30% [73].

Using model systems, researchers studied whether methionine oxidation increases NF-kB (nuclear factor kappa-light-chain-enhancer of activated B) activation, thereby contributing to cerebral ischemia reperfusion injury [74]. In order to have a protective role, methionine must inhibit the NF-kB pathway, since it promotes inflammation. This is because inhibition of this pathway protects against cerebral ischemia reperfusion injury, notably post-ischemic neurovascular inflammation. It was found that the derivative of methionine referred to as methionine sulfoxide reductase (MsrA) protects against hypoxic injury by inhibiting the NF-kB pathway HIV-LTR/Luciferase (HLL) in transgenic male mice. MsrA decreases protein methionine oxidation of calcium/calmodulin-dependent protein kinase II (CaMKII), which partially controls the redox regulation of the NF-kB pathway. Therefore, inhibition of CaMKII results in inhibition of the NF-kB pathway, ultimately mediating brain injury due to hypoxia and reperfusion [74]. For this study, only male mice were used, so it remains unknown what the impact of MsrA is in female mice [74]. Similar results were found regarding the protective effects of MsrA in another study [76]. The focus of this study was to determine whether overexpression of MsrA protects neuronal cells against hypoxia reoxygenation injury. The main findings of this study were that brief hypoxia and reoxygenation cause significant and quick changes in ROS levels in the cells. This increase in ROS causes a depolarization of the mitochondrial membrane of the cells, thereby facilitating cell death and increasing apoptosis. MsrA is an antioxidant that promotes the reduction of met-O in proteins to methionine and, when overexpressed, was found to protect against cellular injuries caused by hypoxia reoxygenation. Overexpression of MsrA lowered the ROS levels in cells caused by hypoxia and reoxygenation, therefore playing a protective role against oxidative stress and promoting cell survival [76]. A strength of this study is that each of the outcomes assessed was a clear indicator of cell death or injury, which allowed researchers to confidently infer the effect of MsrA in protecting against hypoxia and reoxygenation injury. However, this study focused on the effects of hypoxia/reoxygenation only in rat PC12 cells and liver cells. The results of hypoxia and reoxygenation on oxidative status as well as the protective effects of MsrA may be different in other types of cells and in vivo [76].

In another study, researchers looked at the protective effects of methionine sulfoximine (MSO) in middle cerebral artery occlusion as an animal model for stroke [75]. The main findings were that intraperitoneal injection of MSO twenty-four hours before middle cerebral artery occlusion prevented significant infarct volume in the rat cerebral cortex, but not in the basal ganglia. The infarct size of the cerebral cortex was significantly decreased, whereas that in the basal ganglia was not [75]. A strength of this study is that the infarct volume in both the cortex and basal ganglia were measured, instead of only the cortex. This shows that the protective effects of MSO cannot be generalized to all regions of the brain. The study did not determine the specific cause of MSO’s protective effects. This will have to be investigated in future research. In addition, a limitation of this study is that only male Sprague-Dawley rats were used. Therefore, results may differ in female rats [75].

## 5. Transsulfuration

Through the transsulfration pathway, cystathionine β-synthase (CBS) is an enzyme that functions to remove excess homocysteine via direct conversion to cystathionine (Figure 1). CBS acts as the rate-limiting enzyme in the conversion of homocysteine to cystathionine, which is then converted to cysteine, a component of the antioxidant glutathione. Some estimate that upwards of 50% of the cysteine produced in this systemic pathway will be utilized in hepatic glutathione synthesis [77]. There is an increase in glutathione during oxidative stress as shown in mouse cerebral brain tissue. Glutathione can exert its antioxidant abilities, namely the reduction of hydrogen peroxide and lipid peroxides, catalyzed by the enzyme glutathione peroxidase (GPx).

### 5.1. Cystathionine β-synthase (CBS)

An association with CBS expression and hypoxia has been established; when researchers subjected an in vitro model of U87-MG human glioblastoma cells and PC12 pheochromocytoma cells to hypoxic conditions, they reported increased transcription of CBS mRNA and protein synthesis. Application of hypoxia-induced factor (HIF) inhibitors blocked the increases in CBS mRNA and protein synthesis found under hypoxic conditions. The same increases in CBS mRNA and protein synthesis were observed in vivo using Sprague-Dawley rats who were then exposed to hypobaric hypoxia. These results were found to be attenuated by treatment with digoxin, an HIF inhibitor clinically prescribed as an anti-arrhythmic, concurrent with hypobaric hypoxia conditions [78].

More recently, changes of CBS promoter methylation levels in hypertensive and stroke patients as compared to a healthy population. In doing so, promotor methylation was determined to be an independent risk factor for both hypertension and stroke, however, results were only significant in men, and it remains unclear why females did not exhibit the same results. Notably, the study is limited in its population, having only studied participants native to Shenzhen, China [79]. Following the found association of CBS deficiency and vascular thromboembolism, researchers sought to compare proteome alterations in CBS-deficient individuals to those with various subtypes of ischemic stroke (e.g., cardioembolic, lacunar, and large-vessel stroke). The authors concluded that common mechanisms exist between individuals with CBS deficiency and ischemic stroke, particularly cardioembolic stroke, as a result of proteome alterations in molecular networks possessing strong interactions with NFκB. This was found to be particularly affected in acute-phase proteins and blood coagulation. However, results may be influenced by the fact that some of the tested CBS-deficient individuals had a prior history of stroke and thus proteome alterations may have already occurred as a result of the prior ischemia [80].

It is still not fully conclusive whether upregulation of CBS during ischemia is either protective or beneficial to recovery. For example, in a population of 75 patients presenting with premature occlusive arterial disease or occlusive cerebrovascular disease, they were found to have increased levels of homocysteine compared to a normal population. Thus, the authors suppose that CBS deficiency predisposes individuals to occlusive arterial disorders [81]. However, even though its CBS deficiency may be related, its role in the development of stroke is unclear. Interestingly, CBS, via its product cystathionine, has actually been positively associated with risk of stroke. Patients with suspected angina pectoris were followed for a median of 7.3 years. In that time, a linear positive trend was found between plasma cystathionine and stroke risk [82]. Thus, more direct research into the levels of CBS products or enzymatic activity itself is warranted for in vivo research.

Several additional experiments come to the conclusion that either CBS or its downstream products may in fact be negative contributors for risk of ischemic stroke. For example, researchers set out to investigate the association of cysteine, a downstream product of the CBS pathway and upstream substrate of H_2_S production, and its association with impaired outcomes in situations of ischemia and stroke. First, authors analyzed excitotoxic amino acid concentrations (e.g., cysteine, methionine, glutamate, aspartate, and glycine) in 36 patients immediately following clinical diagnosis of stroke by a neurologist. Patients were then stratified into two groups, good outcome or poor outcome, after reassessment at 90 days using the Rankin scale. Plasma cysteine levels provided the most significant association with lower levels resulting in better patient outcomes. The researchers further investigated possible mechanisms using male Wistar rats that were subjected to a middle cerebral artery occlusion (MCAO) concurrent with cysteine administration. Forebrain sections were analyzed for extent of infarct volume. It was found in a dose-dependent manner that cysteine levels were positively correlated with infarct volume. Intraperitoneal administration of aminooxyacetic acid (AOAA), a CBS inhibitor, abolished the cysteine-induced increase in infarct volume. More investigation is necessary since, as the authors point out, rat models demonstrate increased sensitivity to cysteine neurotoxicity and thus infarct volume may be influenced by this factor alone [83]. However, given the efficacy of AOAA in diminishing cysteine-induced infarct volume, it necessitates further investigation into CBS and its role in ischemia. More investigation into CBS, its regulation, and downstream products is necessary. The evidence for the positive direct correlation with CBS activity is becoming clear, however the results warrant more research.

### 5.2. Glutathione

Glutathione is rapidly oxidized in periods of oxidative stress in order to prevent cellular damage as a result of the production of ROS. Rodent [84] and clinical studies [85] have shown that glutathione concentrations are increased after supplementation. In rodents, the increase is seen in thirty minutes, whereas in humans it takes up to one month. Thus, all enzymes related to the synthesis of glutathione pose liabilities in the conditions of ischemia and resulting oxidative stress. As an example of its importance in periods of oxidative stress, researchers found that when male Sprague-Dawley rats were placed in hypoxic conditions with subsequent reoxygenation, significant reductions in glutathione and superoxide dismutase (SOD) were observed [86]. Additionally, as another example of the importance of glutathione, the effects of glutathione peroxidase deficiency under conditions of hyperhomocysteinemia were studied. Investigators found that mice heterozygote for glutathione peroxidase (Gpx^+/−^) fed a high-methionine diet, to recreate conditions of hyperhomocysteinemia, were found to be particularly susceptible to endothelial dysfunction with specific impairment in endothelium-dependent vasodilator function. Moreover, in Gpx^+/−^ mice fed high-methionine diets, acetylcholine aortic ring relaxation was found to be impaired when compared to wild-type mice. Hyperhomocysteinemia was suspected of contributing to this impairment through a peroxide-dependent oxidative mechanism, and GPx deficiency did not impair vascular responses with administration of endothelium-independent vasodilators in either the presence or absence of hyperhomocysteinemia [87]. Thus, GPx reduced susceptibility of endothelial dysfunction during hyperhomocysteinemia conditions through its effects on peroxide oxidative mechanisms.

Glutathione has also been shown to play an important role in periods of ischemia and recovery. Male Sprague-Dawley rats were subjected to transient middle cerebral artery occlusion with or without injection of glutathione 10 min following reperfusion. After 24 h, rats were then analyzed for both brain tissue edema and infarct volume. The experimental control exhibited increased brain tissue edema and infarct volume by 2-fold compared to those with glutathione administration. The glutathione-treated group had more healthy cells in the cortex and striatum compared to control animals. Additionally, analysis of claudin-5, a protein important in the formation of the blood–brain barrier (BBB), revealed a potential role in the protective nature of glutathione following cerebral ischemia. Specifically, models administered glutathione treatment showed increased claudin-5 expression compared to controls. Murine brain endothelial cells were placed in an anaerobic chamber and then administered glutathione or saline. Glutathione resulted in decreased ROS production, as well as increased viability following hypoxia/reperfusion injury. Through Western blot and RT-PCR, the underlying mechanism was found to be associated with increased PI3K/Akt activation as well as forkhead box O3 (FOXO3) inactivation [88]. (See Table 1).

## 6. Conclusions

The in vivo model system studies reviewed in this manuscript used males, therefore the impact of deficiencies in 1C metabolism on stroke in females remains relatively unknown. Women are affected by cardiovascular disease [89], therefore model system studies need to include both sexes in future studies.

1C metabolism is far reaching and impacts several processes in the cell. It plays an important role in metabolizing homocysteine, which is a known risk factor for hypoxic events, such as stroke. Increasing dietary components of 1C has been shown to reduce oxidative stress in animal models. However, more work investigating the clinical applicability is required in order to determine benefits for stroke outcome in patients. Since combinational therapies have been shown to be effective for stroke patients [90], it may be worthwhile to consider incorporating nutrition into a therapeutic plan.

## Figures and Tables

**Table 1:** Summary of main findings of model system studies.

Reference	Model System and Sex Used	Design	Major Findings
[42]	Sprague-Dawley rats (male)	Using focal cerebral ischemia model and folic acid deficiency to investigate neuronal autophagy in neuronal cells.	Autophagy was induced in rats subjected to ischemic insults. Further autophagy activation in cortex neurons caused by folic acid deficiency was confirmed by the increase in the LC3II/LC3I ratio and beclin 1 expression. The increased formation of autophagosomes. results suggest that cerebral cortex cell injury by folic acid deficiency in ischemic brains is partially mediated by the activation of autophagy. Oxidative injury seems to be involved in excessive activation of autophagy caused by folic acid deficiency.
[40]	Mongolian gerbils (male)	Examined whether folic acid deficiency and transient forebrain ischemia enhances neuronal damage and gliosis via oxidative stress in the gerbil hippocampus.	After transient cerebral ischemia Folic acid deficiency increases delayed neuronal death, DNA damage, platelet endothelial cell adhesion molecule 1 immunoreactivity, and gliosis in the hippocampus.
[47]	C57BL/6J mice (male)	Investigated the impact of dietary deficiency of folic acid and supplementation after with folic acid, vitmain B12, riboflavin and choline promoted recovery after photothrombosis ischemic damage.	Combination of B-vitamins, including folic acid, riboflavin and vitamin B12 with choline supplementation promotes some degree of functional improvement following ischemic damage. Additionally, study shows increased neuroplasticity markers deltaFosB and brain derived neurotrophic factor (BDNF), as well as increased levels of nuclear factor erythroid 2–related factor 2 (Nrf2) and superoxide dismutase 2 (SOD2), indicators of anti-oxidant activity
[67]	Wistar Hannover rats (male)	The impact of choline supplementation on the survival of hippocampal neurons following transient forebrain ischemia	There was no difference between choline treated rats up to 200 mg/kg/day and controls (vehicle-treated animals). Choline administered at 400 mg/kg/day provided a significant neuroprotection to ischemic animals at the dose.
[68]	Sprague-Dawley rats (male)	The aim of this study was to determine whether the low-potency and elective alpha-7 neuronal nicotinic cholinergic receptor (nAChR) agonist choline could be a useful treatment for improvement of neurological outcome in a rat model of traumatic brain injury (TBI).	Choline supplemented animals show improved memory retention tests; dietary choline supplementation was associated with cortical tissue preservation; choline supplementation attenuates TBI-induced decreases in cortical levels of alpha-7 nAChR.
[69]	Gerbils (male)	Examined changes and effects of citocoline on phospholipids and glutathione synthesis after transient ischemia and reperfusion. Citcoline is a precursor to choline, which can be metabolized to methionine (1C metabolism), which may be further converted to glutathione, which is one of the primary endogenous antioxidant defense systems in the brain.	The study demonstrated that citicoline supplementation improved phospholipid membrane short-term, while the neuroprotective factors (demonstrated by glutathione) were more significant 3+ days post infarct.
[70]	Sprague-Dawley rats (male)	Investigated whether a chronic treatment with CDP-choline starting 24 h after is middle cerebral artery occlusion in the rat.	Increased neuronal plasticity and contribution to sensorimotor function recovery when chronically treated with CDP-choline.
[74]	C57BL/6J mice (male) and human umbical vein endothelial cells	Study tried to determine if NF-kB (nuclear factor kappa-light-chain-enhancer of activated B) activation is increased by methionine oxidation after ischemic stroke.	The main findings are that using the oxidation of methionine and of CaMMKII Met281/282, reactive oxygen species (ROS) promote the NF-kB pathway in endothelial cells in vitro and in vivo. Furthermore, NF-kB pathway activation and cerebral ischemia/reperfusion injury can be prevented by MsrA, through the expression of MsrA in nonhematopoietic cells. Finally, mice deficient in methionine sulfoxide reductase A (MsrA-/-) have decreased outcome after stroke, but outcome can be protected against by inhibition of the NF-kB pathway or CaMKII.
[75]	Sprague-Dawley rats (male)	The focus of this study was to look at the protective effects of methionine sulfoximine (MSO) in middle cerebral artery occlusion, which was used as an animal model for stroke.	The main findings of this study are that intraperitoneal MSO injection prevented significant infarct volume in the rat cerebral cortex but not the basal ganglia after middle cerebral artery occlusion. MSO administration was found to increase cortical glycogen by 81% 24hours after administration, but did not change glucose levels significantly. Methionine sulfoximine might be changing the presynaptic cells by interrupting the astrocyte-neuron glutamate shuttle and impairing neuronal glutamine synthetase.
[76]	PC12 Cells	To determine whether overexpression of methionine sulfoxide reductase A (MSRA) impacts protection in cells after hypoxia and reoxygenation injury.	The main findings of this study are that brief hypoxia and reoxygenation cause significant and quick changes in reactive oxygen species (ROS) levels in the cells. This increase in ROS causes a depolarization of the mitochondrial membrane of the cells, thereby facilitating cell death, as well as an increase in apoptosis of cells due to the ROS. Methionine sulfoxide reductase type A (MSRA) is an antioxidant that promotes the reduction of met-O in proteins to methionine and, when overexpressed, was found to protect against these cellular injuries caused by hypoxia/reoxygenation. MSRA was found to lower the ROS levels in cells caused by hypoxia/reoxygenation therefore playing a protective role against oxidative stress causing cell injury.
[78]	Cells: U87-MG, PC12, human lung microvascular and aortic endothelial cells, and primary vascular smooth muscle Sprague-Dawley Wistar rats cell cultures	Study examined the relationship of hypoxia and expression of cystathionine beta synthase (CBS).	mRNA and protein expression of CBS were increased after hypoxia. The increase may be mediated by hypoxia-inducible factors (HIFs) in the cell models.
[86]	Sprague-Dawley rats (male)	The study evaluate the effects of exposition to acute severe repiratory hypoxia followed by reoxygenation in brain injury.	After hypoxia and reoxygenation, oxidative stress and apoptosis were increased.
[88]	Sprague-Dawley rats (male) and endothelial cells	The study examined whether glutathione reduces cerebral infarct size after middle cerebral artery occulsion	In vivo GSH-deficiency resulted in increased cerebral infarction volume. GSH reduced brain infarct volume. the expression of claudin-5 associated with brain infarct formation.We also examined activation of the PI3K/Akt pathway, inactivation of FOXO3, and expression of Bcl2 to assess the role of GSH in promoting cell survival in response to ischemic injury.
